# Periostin is up-regulated in high grade and high stage prostate cancer

**DOI:** 10.1186/1471-2407-10-273

**Published:** 2010-06-09

**Authors:** Verena Tischler, Florian R Fritzsche, Peter J Wild, Carsten Stephan, Hans-Helge Seifert, Marc-Oliver Riener, Thomas Hermanns, Ashkan Mortezavi, Josefine Gerhardt, Peter Schraml, Klaus Jung, Holger Moch, Alex Soltermann, Glen Kristiansen

**Affiliations:** 1 Institute for Surgical Pathology, University Hospital Zurich, Zurich, Switzerland; 2 Department of Urology, University Hospital Zurich, Zurich, Switzerland; 3 Department of Urology, Charité - Universitätsmedizin, Berlin, Germany; 4 Berlin Institute for Urologic Research, Charité - Universitätsmedizin, Berlin, Germany

## Abstract

**Background:**

Expression of periostin is an indicator of epithelial-mesenchymal transition in cancer but a detailed analysis of periostin expression in prostate cancer has not been conducted so far.

**Methods:**

Here, we evaluated periostin expression in prostate cancer cells and peritumoural stroma immunohistochemically in two independent prostate cancer cohorts, including a training cohort (n = 93) and a test cohort (n = 325). Metastatic prostate cancers (n = 20), hormone refractory prostate cancers (n = 19) and benign prostatic tissues (n = 38) were also analyzed.

**Results:**

In total, strong epithelial periostin expression was detectable in 142 of 418 (34.0%) of prostate carcinomas and in 11 of 38 benign prostate glands (28.9%). Increased periostin expression in carcinoma cells was significantly associated with high Gleason score (p < 0.01) and advanced tumour stage (p < 0.05) in the test cohort. Whereas periostin expression was weak or absent in the stroma around normal prostate glands, strong periostin expression in tumour stroma was found in most primary and metastatic prostate cancers. High stromal periostin expression was associated with higher Gleason scores (p < 0.001). There was a relationship between stromal periostin expression and shortened PSA relapse free survival times in the training cohort (p < 0.05).

**Conclusions:**

Our data indicate that periostin up-regulation is related to increased tumour aggressiveness in prostate cancer and might be a promising target for therapeutical interventions in primary and metastatic prostate cancer.

## Background

Periostin (POSTN) is a 93 kDa N-glycoprotein, first described in 1993 in mouse osteoblasts as osteoblast-specific factor 2 (OSF-2). It shows homology with the cell adhesion molecules fasciclin 1 (drosophila) and beta-IgH3 (human), sharing features that are thought to explain some of its functional characteristics [1,2] like involvement in cell adhesion and osteoblast recruitment [3].

Periostin has been found in several, mainly collagen-rich and fetal tissues as an extracellular matrix protein and is up-regulated by mechanical stress during tissue repair and (re)generation [4-8]. Periostin expression can be induced by vascular injury which in turn induces vascular endothelial growth factor receptor 2 with consequent promotion of angiogenesis [9,10]. After myocardial infarction, periostin up-regulation seems to be important for the healing process [11,12].

As a ligand to alpha(V)beta(3) and alpha(V)beta(5) integrin periostin appears to activate the Akt/PKB (protein kinase B) pathway, known to facilitate cell survival and tumourigenesis [13-15].

High expression of periostin protein or mRNA was detected in most solid tumours including breast, colon, head and neck, pancreatic, papillary thyroid, ovarian, lung, gastric and liver carcinoma, as well as neuroblastoma [9,13,16-33]. As periostin is a secreted protein, it is not surprising that elevated periostin levels in serum and pleural effusion have recently been detected in lung cancer patients [28,34]. Suggested effects of periostin on tumour cells include increased growth and resistance against hypoxia and chemotherapeutics [16,17].

So far there is only a single report on periostin expression in prostate cancer [35]. Increased cancer cell expression of periostin compared to normal glands was found during early stages of prostate cancer whereas in advanced stages stromal periostin expression prevailed [35]. The aim of our study was to determine the periostin expression in the stromal and epithelial compartment of the tumour, as well as the correlation with clinical data including patient follow up data in a larger cohort.

## Methods

### Patients

A training cohort was used for the establishment of a periostin evaluation algorithm. The training cohort consisted of tissue of 93 prostate cancer patients diagnosed between 1990 and 2001 at the Institute of Pathology, Charité - Universitätsmedizin Berlin. In this cohort cases with and without PSA relapse were selectively chosen to study the relevance of biomarkers for prediction of PSA relapse. The median age was 61 years (range 47-73 years). The pT-status was pT2 in 42 (45.2%) and pT3/4 in 51 (54.8%) cases. The Gleason score was < 7 in 23 (24.7%), 7 in 39 (41.9%) and >7 in 31 (33.3%) cases. Forty-one (44.1%) tumours were judged R1, 50 (53.8%) R0 and 2 (2.1%) Rx. Forty-three (46.2%) patients had a PSA relapse. The median follow-up time was 45 months (range 3-180 months).

In a second step, periostin expression was analyzed in a larger test cohort with 325 primary prostate cancers. The test cohort consisted of 325 consecutive patients treated with prostatectomy for prostate cancer between 1993 and 2006 at the Department of Urology, University Hospital Zurich. The median age was 64 years (range 46-79 years). The pT-status was pT2 in 205 (63.1%) and pT3/4 in 120 (36.9%) cases. The Gleason score was < 7 in 50 (15.4%), 7 in 194 (59.7%) and >7 in 81 (24.9%) tumours. Concerning surgical margins, 112 (34.5%) tumours were R1, 207 (63.7%) R0 and 6 (1.8%) Rx. Sixty-eight (20.9%) patients had a PSA relapse. The median follow-up time was 72 months (range 0-163 months). Data on relapse free survival times was available for 211 of the patients. In addition 20 metastatic prostate cancers (organ metastasis; 19 bone metastasis and 1 bladder metastasis), 19 hormone resistant prostate cancers and 38 cases of benign prostatic tissue were evaluated. The 19 hormone resistant prostate cancer specimens were from patients undergoing palliative transurethral prostate resection in advanced disease.

The study was approved by the the Charité University Ethics Committee (EA1/06/2004) and by the Cantonal Ethics Committee of Zurich (StV 25-2007 neu). In the latter, necessity of patients' informed consent was explicitely ruled out, since this is a retrospective study.

### Tissue microarray construction

The tissue microarrays (TMA) were constructed as previously described [36]. We used commercially available tissue arrayers (Beecher Instruments, Woodland, CA, USA) and applied a core diameter of 0.6 mm for the tissue samples of Zurich and 1.0 mm for the tissue samples of Berlin. Each tumour was represented by one tissue core.

### Immunohistochemistry (IHC)

The TMA blocks were freshly cut (3 μm) and mounted on superfrost slides (Menzel Gläser, Braunschweig, Germany). IHC was conducted with the Ventana Benchmark automated staining system (Ventana Medical Systems, Tucson, AZ, USA) using Ventana reagents and a polyclonal antibody against human periostin (OSF-2/periostin, BioVendor Laboratory Medicine, Modrice, Czech Republic; RD181045050 RD-932, 1:500) after standard (CC1 m) heat induced antigen retrieval as described before [32]. The antibody dilution was titrated using small test arrays as described elsewhere [32]. Detection was performed using the UltraVIEW™ DAB detection kit.

### Evaluation of stainings

The periostin protein expression was evaluated by two clinical pathologists (FRF, GK) on a multi-headed microscope. The test cohort was subsequently re-evaluated by another pathologist (AS).

For evaluation of epithelial and stromal periostin expression, we implemented an immuno-reactive score including intensity and quantity of cells stained. The staining intensity was scored negative (0), weak (1+), moderate (2+) or strong (3+). The quantity of stained cells was scored zero (0), < 10% (1+), 10-50% (2+), 51-80% (3+) or >80% (4+). Intensity and quantity were multiplied (immunoreactive score (IRS), range 0-12).

### Statistical analysis

Statistical analyses were performed with SPSS 17.0 (SPSS Inc., Chicago, IL, USA). The median value of the IRS was used as cut-off point to dichotomize the tumours into a "periostin *low" *and "periostin *high" *group. Fisher's exact and chi-squared tests were applied to assess associations between categorized periostin expression and clinico-pathological parameters. Correlations were computed using Spearman's bivariate rank order correlation. Univariate survival analysis was carried out according to Kaplan-Meier, differences in survival curves were assessed with the Log rank test. P-values < 0.05 were considered significant.

## Results

### Periostin expression in epithelia and stroma of prostate tissues

**Figure 1:**
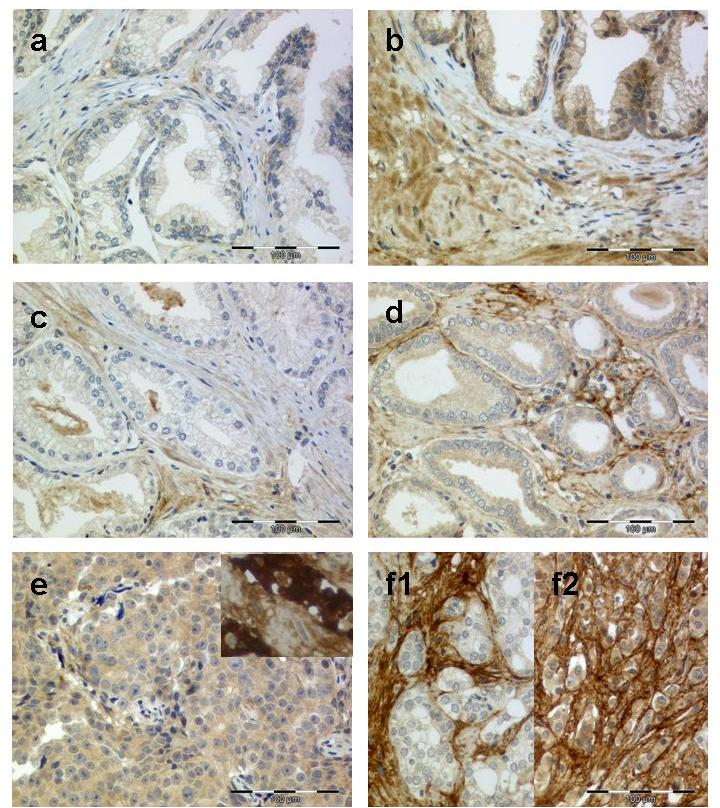
**Periostin protein expression in malignant and benign prostate tissue**. Weak (**a**) to moderate (**b**) epithelial and negative (**a**) to weak (**b**) stromal periostin expression in benign prostate glands. In contrast to most cancer cases, in benign tissue with stromal periostin expression the direct periglandular area is rather negative. Some cases showed a weak periostin positivity of basal cells. Prostate cancers with negative (**c**), weak (**d**), moderate (**e/f2**) and strong (**e **inset) epithelial periostin expression. The peritumoural stroma was weakly (**c**), moderately (**d**) or strongly (**f1/f2**) positive.

Distinct stromal and epithelial staining characteristics allowed an absolutely certain evaluation of the periostin staining (Figure 1a and 1b). Benign prostate glands expressed high stromal periostin in only 2/38 cases and high epithelial periostin in 11/38 cases. From the 38 benign prostate samples, 24 displayed no periglandular stromal and 14 no epithelial periostin expression. Of the 24 benign cases without stromal periostin expression, 19 showed epithelial periostin expression and vice versa of the 14 benign cases without periostin expression, 9 revealed stromal periostin expression. Both periostin epithelial and stromal negativity occurred in 5 of the benign cases. Five cases were positive for periostin in both epithelia and stroma. Basal cells showed in some cases a slightly stronger staining than the inner secretory cell layer. In the remaining tumour epithelia, periostin was detected in the cytoplasm without luminal or membranous accentuation. Nuclear staining was not observed. The stroma displayed a fibrillary pattern with considerable variation of intensity. Staining intensity differed frequently within a respective case between the stroma and epithelium.

Tumour stroma was positive in all cases of the training cohort and most cases of the test cohort. Only 11 (3.4%) cases of the test cohort were negative. An IRS above 2 was found in the vast majority of primary (82.8%), hormone resistant (78.9%) and metastatic (85%) tumours. Using our periostin score, it was possible to differentiate between low and high periostin expression levels using the median. The median stromal IRS for both primary prostate cancer cohorts was 6 with a mean value of about 5.5.

Sixty (18.5%) primary prostate cancer cases showed no epithelial periostin expression and 189 (58.2%) cases had an IRS equal to or below 3 (median 3). In total, 142/418 prostate cancer cases expressed high levels of epithelial periostin. Only 7.4% of cases exhibited an IRS for epithelial periostin expression above 6. Revalidation of the stainings resulted in the same median IRS values.

For the 19 hormone resistant prostate cancers the median IRS was 8 for the stromal and 4 for the epithelial periostin expression (means: 6.8 and 5.1). In the 20 samples from prostate cancer metastases the mean and median IRS of epithelial and stromal periostin expression did not differ from that in the primary prostate cancers.

### Correlations and associations with clinico-pathological parameters

In the training cohort, stromal periostin expression showed no correlation (Spearman rank order) with any of the clinico-pathological parameters (age, pT-status, Gleason score, residual status). A higher pT stage was significantly associated with high epithelial periostin expression (p = 0.026, Table 1). However, Fisher's exact test revealed a significant association of higher periostin stromal expression with positive resection margins (R1) (high periostin expression in R0 versus R1: 14% (n = 7) versus 39% (n = 16); p = 0.008). No other associations were detected (Table 1). In the test cohort, high stromal and epithelial periostin expression were both associated with high Gleason scores (p = 0.011 and 0.007, Table 2). For epithelial expression an additional significant association with advanced pT-status was demonstrated (p = 0.048, Table 2). In the Spearman rank order correlation for the test cohort, the significant associations from above could be confirmed for epithelial expression (p-values: 0.001 and 0.047, Table 3). For stromal expression, the correlation with Gleason score was also significant (p = 0.003, Table 3). Stromal periostin expression was significantly correlated with epithelial periostin expression (p = 0.003, Table 3).

**Table 1:** Stromal and epithelial periostin expression in prostate cancer and clinico-pathological parameters of the training cohort

	Total n (%)	Periostin stromal low n (%)	Periostin stromal high n (%)	Periostin epithelial low n (%)	Periostin epithelial high n (%)	p-values stromal/epithelial
**All cases**	93(100)	45 (48.4)	48 (51.6)	87 (93.5)	6 (6.5)	

**Age**						0.905/ 0.484
≤ 64	49 (52.7)	24 (49.0)	25 (51.0)	45 (91.8)	4 (8.2)	
>64	44 (47.3)	21 (47.7)	23 (52.3)	42 (95.5)	2 (4.5)	

**pT-status**						0.573/**0.026**
pT2	42 (45.2)	22 (52.4)	20 (47.6)	42 (100)	0 (0)	
pT3/4	51 (54.8)	23 (45.1)	28 (54.9)	43 (84.3)	6 (11.7)	

**Gleason score**						0.523/ 0.404
3-6	23 (24.8)	13 (56.5)	10 (43.5)	22 (95.7)	1 (4.3)	
7	39 (41.9)	19 (48.7)	20 (51.3)	38 (97.4)	1 (2.6)	
8-10	31 (33.3)	13 (41.9)	18 (58.1)	27 (87.1)	4 (12.9)	

**Residual tumour^a^**						0.322/ 0.052
R0	50 (54.9)	26 (52.0)	24 (48.0)	49 (98.0)	1 (0.02)	
R1	41 (45.1)	17(41.5)	24 (58.5)	36 (87.8)	5 (12.2)	

^a^Two cases were Rx

**Table 2:** Stromal and epithelial periostin expression in prostate cancer and clinico-pathological parameters of the test cohort

	Total n (%)	Periostin stromal low n (%)	Periostin stromal high n (%)	Periostin epithelial low n (%)	Periostin epithelial high n (%)	p-values stromal/epithelial
**All cases**	325 (100)	224 (68.9)	101 (31.1)	189 (58.2)	136 (41.8)	

**Age**						0.120/ 1.000
≤ 64	165 (50.8)	107 (64.8)	58 (35.2)	96 (58.2)	69 (41.8)	
>64	160 (49.2)	117 (73.1)	43 (26.9)	93 (58.1)	67 (41.9)	

**pT-status**						0.710/**0.048**
pT2	205 (63.1)	143 (69.8)	62 (30.2)	128 (62.4)	77 (37.6)	
pT3/4	120 (36.9)	81 (67.5)	39 (32.5)	61 (50.8)	59 (49.2)	

**Gleason score**						**0.011**/ **0.007**
3-6	50 (15.4)	40 (80.0)	10 (20.0)	35 (70.0)	15 (30.0)	
7	194 (59.7)	136 (70.1)	58 (29.9)	116 (59.8)	78 (40.2)	
8-10	81 (24.9)	48 (59.3)	33 (40.7)	38 (46.9)	43 (53.1)	

**Residual tumour^a^**						0.451/ 0.634
R0	207 (63.7)	139 (67.1)	68 (32.9)	119 (57.5)	88 (42.5)	
R1	112 (34.5)	80 (71.4)	32 (28.6)	68 (60.7)	44 (39.3)	

^a^Six cases were Rx

**Table 3:** Periostin protein expression (stromal and epithelial) with clinico-pathological parameters in the test cohort

Periostin	Periostin stromal	Periostin epithelial	pT-status	Gleason sum	Age	Residual tumour
Periostin epithelial						
**CC**		0.162	0.057	0.162	-0.108	-0.020
**p-value**		**0.003**	0.307	**0.003**	0.053	0.720
**Number of cases**		325	325	325	325	319

Periostin epithelial						
**CC**	0.162		0.110	0.191	-0.056	-0.027
**p-value**	**0.003**		**0.047**	**0.001**	0.313	0.626
**Number of cases**	325		325	325	325	319

CC = Correlation coefficient

### Periostin and PSA relapse free survival

**Figure 2:**
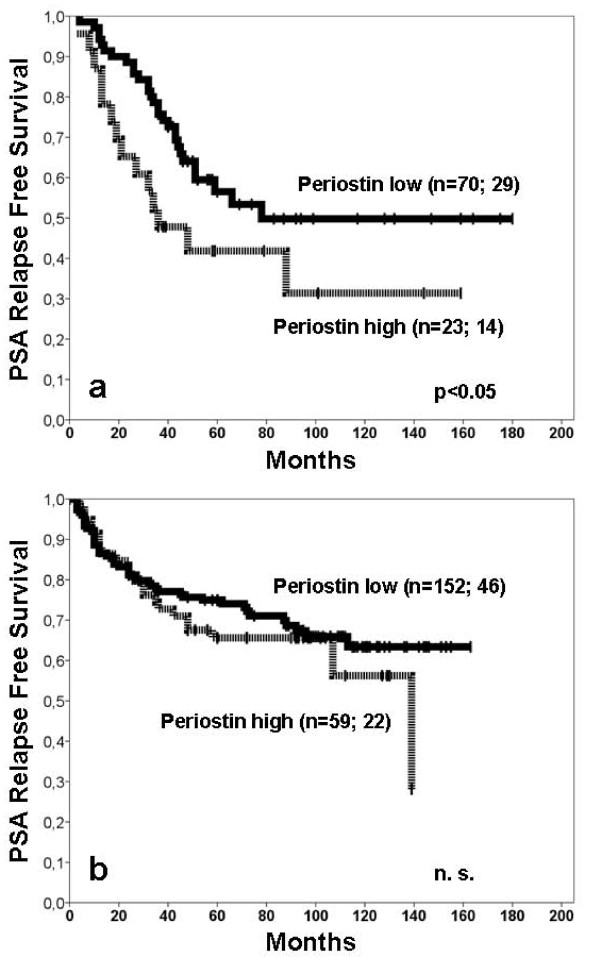
**PSA relapse free survival for periostin in training and test cohort**. **a) **In the training cohort higher stromal periostin expression was a significant prognosticator for shortened PSA relapse free survival (p = 0.045). The periostin *low *group consisted of 70 patients of which 29 had a PSA relapse. In the periostin *high *group 14 of the 23 patients had a PSA relapse. **b) **In the test cohort the curve of those patients with higher stromal periostin expression remained slightly below that of the patients with lower stromal periostin expression (p = 0.373). In the test cohort the periostin *low *group consisted of 152 patients. Forty-six patients had a PSA relapse (periostin *high *group: 22 of the 59 patients with PSA relapse).

The standard prognosticators (pT-status, Gleason score and residual tumour) correlated significantly with shortened PSA relapse free survival in both cohorts (Table 4, training cohort not shown). In the training cohort, high stromal periostin was significantly associated with shortened PSA relapse free survival times (p < 0.05; Figure 2a).

**Table 4:** PSA relapse free survival in dependence of stromal and epithelial periostin expression and clinico-pathological parameters in the test cohort

Characteristic	No. of cases	No. of events	3-year PSA relapse rate (± SE) in %	p-values
**Periostin stromal**				0.373

low	152	46	77.1 ± 3.5	
high	59	22	72.7 ± 5.8	

**Periostin epithelial**				0.722

low	133	41	77.7 ± 3.6	
high	78	27	72.6 ± 5.1	

**Age**				0.514

≤ 64 years	108	33	79.2 ± 4.0	
>64 years	103	35	74.3 ± 4.3	

**pT status**				**< 0.001**

pT1/2	153	37	84.2 ± 3.0	
pT3/4	58	31	56.4 ± 6.7	

**Gleason score**				**< 0.001**

3-6	46	4	93.2 ± 3.8	
7	131	44	76.0 ± 3.8	
8-10	34	20	52.0 ± 8.7	

**Residual tumour^a^**				**< 0.001**

R0	151	34	83.8 ± 3.0	
R1	59	33	60.4 ± 6.4	

^a^One case was Rx.

In the test cohort, neither stromal nor epithelial periostin expression reached prognostic significance (relapse free survival, p = 0.373 respectively p = 0.722) (Figure 2b).

## Discussion

In this study, we provide evidence for periostin up-regulation during prostate cancer progression. Periostin expression was found in both epithelial cancer cells and in peritumoural stroma. Recently, our group has demonstrated that periostin as a marker for the epithelial-mesenchymal-transition (EMT) programme in lung cancer is prognostically relevant [32]. EMT is correlated with tumour progression and represents an important form of tumour-stroma interaction facilitating the stromal invasion of the cancer cells. Periostin seems to play an important part in this prognostically adverse transdifferentiation process. However, the regulation mechanisms of periostin in tumour progression have not been elucidated so far. Our data demonstrate a significant association between periostin and pT-stage, Gleason grade and involvement of prognosis in two different prostate cancer cohorts, suggesting that EMT is of utmost importance for prostate cancer progression. There is only one study by Tsunoda *et al*. observing a prostate cancer patient cohort of 77 prostate cancers showing increased periostin expression in early prostate cancer stages as well as in the stroma of advanced prostate cancer cases [35]. This is in contrast to our study of 418 prostate carcinomas where we find increased epithelial periostin expression positively correlated to grade and stage and increased stromal periostin positively correlated to grade. Augmentation of both epithelial and stromal periostin in our cohort is a characteristic of the advanced and more aggressive prostate cancer cases. This observation is further supported by the finding that only 2/38 benign prostate tissues expressed stromal periostin. The differences of Tsunoda *et al*. and our results may be explained by the sample number (77 versus 418) and differences in grade and stage. Our test cohort was represented by 63.1% pT2 and 36.9% pT3/4 tumours whereas Tsunoda's cohort comprised of 18.2% pT2 and 74.0% pT3/4 tumours. Grade in our test cohort was < 7 in 15.4%, 7 in 59.7% and >7 in 24.9%. Tsunoda's cohort had a much higher percentage of Gleason 8-10 of 55.8%. Altogether, our test cohort might be more representative for early stages of prostate cancer than Tsunoda's which could well explain the observed difference.

We do acknowledge that a core diameter of 0.6 mm per prostate cancer case might not be fully representative for a given case. This is especially of importance in small cohorts. However, during the preparation of our tissue microarray, we reviewed each case very carefully to choose a representative area of tumour tissue.

There are several reports on involvement of other EMT markers eg. platelet-derived growth factor-D, hypoxia-inducible factor-1α and zinc finger enhancer binding protein 1 in prostate cancer [37-40]. Meanwhile, periostin has been found up-regulated in several tumour entities, either in the stroma, the epithelial cells or in the serum [17,26,27,30,41,42]. In most of these tumour entities, periostin has been associated with more aggressive tumour characteristics, which is perfectly in line with our findings in prostate cancer. Apart from EMT, periostin is related to other stromal re-modeling and repair processes such as wound healing or formation of heart valves in embryogenesis. It is not clear yet whether periostin upregulation reflects only the stroma re-modeling process *per se *or whether it is actively induced by the tumour cells themselves. The presence of both mRNA and protein in the cytoplasm of tumour cells favours an active or signal transducting role of periostin, respectively. Further functional studies are needed to shed light on the mechanism of periostin up-regulation in prostate cancer.

In our test cohort we could not reproduce the promising results concerning the prognostic value of periostin deduced from the training cohort. A possible explanation is most likely the selection of the training cohort with a very high number of cases with PSA relapse (46%) whereas the consecutive cases of the test cohort show usual relapse rates (21%). The selection of the training cohort was done to identify biomarker for PSA relapse. It is not uncommon to see that prognostic significances are better in trainings cohorts than in tests cohorts. The differing results for relapse free survival of both cohorts are therefore not too surprising for us, also taking into account that the composition of the cohorts is so different. However, this also demonstrates that the prognostic value of periostin is limited in comparison to well established conventional prognosticators of prostate cancer and other potentially prognostic molecular markers [36,43]. More important than a prognostic value of periostin might be its use as a therapeutic target. Kudo *et al*. and Castranovo *et al*. have recently evaluated its therapeutic potential [41,44]. In a chemical proteomics approach, periostin was found accessible by the blood stream, which is an important factor for effective drugability [41,44]. It has been concluded that its expression characteristics and cancer specific up-regulation make periostin a promising target for ligand-based tumour targeting strategies. Considering its high expression in both stroma and tumour cells, this might be an auspicious option for advanced prostate cancer. Also, the diagnostic serological value of periostin might be worth looking at.

## Conclusions

This immunohistochemical study describes the periostin protein expression pattern in prostate cancer and benign prostate tissue in a large patient cohort. Its upregulation in primary, metastatic and hormone resistant prostate cancers was related to a more aggressive and advanced tumour biology. These expression characteristics and its proposed drugability make periostin a promising target for an individualized prostate cancer therapy.

## Competing interests

The authors declare that they have no competing interests.

## Authors' contributions

HM, AS, GK and VT designed the study, participated in the statistical analysis and drafted the manuscript. FRF and JG participated in the design of the study and performed the statistical analysis. PJW, PS, TH, HHS and HM designed the "Zurich" tissue microarray. GK, FRF, KJ and CS designed the "Berlin" tissue micro array. HHS, PJW, AM and MOR participated in collecting clinical data for the "Zurich" cohort. HM, GK, AS conceived the study, participated in its design and coordination and helped to draft the manuscript. All authors read and approved the final manuscript.

## Pre-publication history

The pre-publication history for this paper can be accessed here:

http://www.biomedcentral.com/1471-2407/10/273/prepub
